# Physical environmental factors related to walking and cycling in older adults: the Belgian aging studies

**DOI:** 10.1186/1471-2458-12-142

**Published:** 2012-02-23

**Authors:** Jelle Van Cauwenberg, Peter Clarys, Ilse De Bourdeaudhuij, Veerle Van Holle, Dominique Verté, Nico De Witte, Liesbeth De Donder, Tine Buffel, Sarah Dury, Benedicte Deforche

**Affiliations:** 1 Department of Human Biometry and Biomechanics, Faculty of Physical Education and Physical Therapy, Vrije Universiteit Brussel, Pleinlaan 2, B-1050 Brussels, Belgium; 2 Department of Movement and Sport Sciences, Faculty of Medicine and Health Sciences, Ghent University, Watersportlaan 2, B-9000 Ghent, Belgium; 3 Department of Adult Educational Sciences, Faculty of Psychology and Educational Sciences, Vrije Universiteit Brussel, Pleinlaan 2, B-1050 Brussels, Belgium; 4 University College Ghent, Keramiekstraat 78-80, B-9000 Ghent, Belgium; 5 Fund for Scientific Research Flanders Belgium, Egmontstraat 5, B-1000 Brussels, Belgium

## Abstract

**Background:**

Socio-ecological models emphasize the relationship between the physical environment and physical activity (PA). However, knowledge about this relationship in older adults is limited. Therefore, the present study aims to investigate the relationship between area of residence (urban, semi-urban or rural) and older adults' walking and cycling for transportation and recreation. Additionally, relationships between several physical environmental factors and walking and cycling and possible moderating effects of area of residence, age and gender were studied.

**Methods:**

Data from 48,879 Flemish older adults collected in 2004-2010 through peer research were analyzed. Walking, cycling and environmental perceptions were assessed using self-administered questionnaires. The Study Service of the Flemish Government provided objective data on municipal characteristics. Multilevel logistic regression analyses were applied.

**Results:**

Urban participants were more likely to walk daily for transportation compared to rural (OR = 1.43; 95% CI = 1.22, 1.67) and semi-urban participants (OR = 1.32; 95% CI = 1.13, 1.54). Urban participants were less likely to cycle daily for transportation compared to semi-urban participants (OR = 0.72; 95% CI = 0.56, 0.92). Area of residence was unrelated to weekly recreational walking/cycling. Perceived short distances to services (ORs ranging from 1.04 to 1.19) and satisfaction with public transport (ORs ranging from 1.07 to 1.13) were significantly positively related to all walking/cycling behaviors. Feelings of unsafety was negatively related to walking for transportation (OR = 0.93, 95% CI = 0.91, 0.95) and recreational walking/cycling (OR = 0.95, 95% CI = 0.92, 0.97). In females, it was also negatively related to cycling for transportation (OR = 0.94, 95% CI = 0.90, 0.98).

**Conclusions:**

Urban residents were more likely to walk for transportation daily compared to semi-urban and rural residents. Daily cycling for transportation was less prevalent among urban compared to semi-urban residents. Access to destinations appeared to be important for promoting both walking and cycling for transportation and recreation across all demographic subgroups. Additionaly, feelings of unsafety were associated with lower rates of walking for transportation and walking/cycling for recreation in all subgroups and cycling for transportation in females. No clear patterns emerged for other environmental factors.

## Background

Engaging in moderate-intensity physical activities (PA) for 30 minutes on five days a week significantly reduces the age-related risk of chronic disease in the growing population of older adults (≥ 65 years) [1,2]. However, 60-70% of older adults do not meet this recommendation [3,4].

Walking and cycling are appropriate activities to promote in older adults as they are both safe, accessible and easy to implement into one's daily routine (e.g. walking to a shop). In order to design effective interventions, knowledge of correlates of walking and cycling is required [5]. Currently, most researchers adopt a socio-ecological framework to explain PA behaviors, with attention for the physical environment [6-8]: the objective and perceived characteristics of the physical context in which people spend their time (e.g. neighborhood), including aspects of urban design (e.g. residential density), traffic density and speed, distance to and design of venues for PA (e.g. parks), crime and safety [9]. Physical environmental characteristics relate differently to walking/cycling for transportation and recreation [10]. In their review, Saelens and Handy [11] found residential density, land use mix and proximity of nonresidential destinations being consistently related to walking for transportation, while recreational walking seemed more closely related to pedestrian infrastructure.

Area of residence (urban, semi-urban or rural) might also relate to walking and cycling as these areas differ in residential densities, proximity of destinations, traffic, greenery... To our knowledge only two studies previously focused on the relationship between urban versus semi-urban residence and older adults' walking for transportation. Su and colleagues [12] found living in the inner city of London to be related to less walking for transportation, whilst Borst and colleagues [13] reported no relationship in the Netherlands. A concept explaining possible relationships between area of residence and walking for transportation is walkability (a composite score of residential density, land use mix and street connectivity) as urban areas will generally be classified as more walkable compared to rural areas. Walkability has been found to relate positively to transportation walking [14] and a combined measure of transportation walking and cycling [15] in US older adults. No previous studies investigated the relationship between area of residence and older adults' recreational walking, recreational cycling or cycling for transportation.

A recent review showed that previous studies yielded inconsistent results and concluded that knowledge concerning the relationship between the physical environment and older adults' walking for transportation and recreation and its moderators is limited. Furthermore, the included studies were predominantly conducted in US urban areas and their findings are not necessarily translatable to other contexts [16]. Finally, only two previous studies focused on the relationship between the physical environment and older adults' cycling [15,17]. Nevertheless, the physical environment might be especially relevant for older adults as they might be more susceptible to physical barriers (e.g. distances, slopes, obstacles...) because of functional limitations and associated fear of moving outdoors [18-20].

Consequently, the primary objective of the present study is to investigate the relationship between area of residence (urban, semi-urban or rural) and walking and cycling for transportation and recreation in Flemish older adults. Second, the relationship between several physical environmental factors and walking and cycling and possible moderating effects of area of residence, age and gender will be studied.

## Methods

### Sampling and data collection

Detailed information on data collection of the Belgian Aging Studies (BAS) has been reported previously [21,22]. Briefly, persons aged 60 years or older were randomly sampled, stratified for age and gender, within participating municipalities. The project of the Belgian Ageing Studies used a participatory data collection methodology, namely peer research. Older adults were involved in the study, not only as the research target group, but they adopted the role of researchers by delivering and collecting questionnaires in their peer group. The questionnaire was meant to be self-administered, but volunteers were allowed to clarify the meaning of questions, when this was requested. The older volunteers were recruited within their municipalities and received several training sessions. In each municipality, on average between 30 and 50 volunteers participated in the project. Peer research has the advantages of face-to-face research (which has a higher response rate), while minimizing the social desirability. Furthermore, it results in more complete questionnaires and a high response rate [23]. In the present study, first response rates ranged from 65 to 85%, depending on municipality. 63,812 community-dwelling persons within 135 of the 308 Flemish municipalities agreed to participate. Data collection was performed between 2004 and 2010.

For the present study, respondents aged < 65 years were excluded, resulting in a final sample of 48,879 older adults within 135 municipalities (36 urban, 50 semi-urban and 49 rural municipalities). The study was approved by the ethical committee of the hospital of the Vrije Universiteit Brussel (B.U.N. 143201111521).

### Measures

*Level of education *was assessed by the following question: 'What is the highest degree of education that you have obtained?' A 10-item response scale ranging from 'no degree' to 'university degree' was provided. Responses were dichotomized into 'no tertiary education' versus 'tertiary education'.

*Functional limitations *were measured by 7 items of the 'physical functioning' subscale of the validated SF-36 [24,25]. Activities in which participants reported to be limited were summed to obtain 'number of functional limitations'.

*Walking for transportation *was measured by: 'How often do you walk for transportation?' Respondents answered on a 5-point scale ranging from 'never' to 'daily'. Scores were dichotomized into daily walking for transportation versus less than daily walking for transportation. C*ycling for transportation *was assessed similarly.

*Walking or cycling for recreation *was measured by a single-item question: 'How often do you walk or cycle for recreation?' A 5-point scale was provided ranging from 'never' to 'more than once a week'. Scores were dichotomized into weekly versus less than weekly walking or cycling for recreation.

*Perceived *short distances to services was assessed through: 'How applicable is the following statement to your neighborhood? Services are located within short distances from my home.' A 5 point-scale ranging from 'completely not applicable' to 'completely applicable' was provided. Absence of high ramps was measured through a similar question. Perceived presence of different kinds of shops, walking facilities and street lighting were measured by a single-item question: 'Are the following facilities sufficiently present in your neighborhood?' Answer categories were 'yes' or 'no'. Responses on answers concerning the presence of different kinds of shops were summed to create the variable 'number of shops'. Satisfaction with public transportation, the condition of sidewalks and satisfaction with greenery were assessed as follows: 'How satisfied are you with following services?'. A 5-point scale ranging from 'completely unsatisfied' to 'completely satisfied' was provided. Traffic safety was measured by the question: 'How often have you experienced problems with unsafety in traffic?' A 4-point scale was provided ranging from never to often and answers were dichotomized into 'seldom or not experiencing problems' and 'experiencing problems'. Personal safety was measured by the 'Elderly Feelings of Unsafety' (EFU) scale. A higher score on this scale reflects a higher degree of feelings of unsafety. This 8-items scale was developed and validated by De Donder and colleagues [21] using data from BAS. In the present sample, internal consistency was found to be good (Cronbach's alpha = 0.84). Information on absence of decay and noise was obtained through the question: 'Which of the following conditions are applicable to your neighborhood?'. Answer categories were 'yes' or 'no'.

*Objective *data on municipalities' residential densities, annual incomes and % public transportation subscriptions in 2008 were obtained from the Study Service of the Flemish Government (available on http://aps.vlaanderen.be/lokaal/lokale_statistieken.htm). Municipalities were categorized as follows: rural when residential density ≤ 150 inhabitants/km^2^, semi-rural when density is between 150 and 300 inhabitants/km^2^, semi-urban when density is between 300 and 600 inhabitants/km^2 ^and urban when density > 600 inhabitants/km^2 ^[26]. As rural areas are scarce in Flanders, rural and semi-rural areas were collapsed into one category 'rural areas'.

### Analyses

To control for clustering of data within municipalities, multi-level analyses were conducted using the MLwiN 2.23 software. Logistic regression was used to predict odds of daily walking for transportation, daily cycling for transportation and weekly walking or cycling for recreation. Model parameter estimates were obtained via Markov Chain Monte Carlo (MCMC) procedures. First, relationships with demographics, number of functional limitations and area of residence were calculated within the total sample using a multivariate model. Secondly, first, second and third order interaction effects with urbanization, age category and gender were analyzed for each environmental factor. If none of the interaction effects were significant the main effect of this environmental factor was analyzed in the total sample. Main effects for environmental factors with significant third, second or first order interaction effects will be presented consecutively in the corresponding subgroups. In case of significant third order interaction effects, no second and first order interaction effects were presented. In case of significant second order interaction effects, no first order interaction effects were presented. All analyses were controlled for level of education and number of functional limitations. Significance level was set at 0.05.

## Results

### Descriptive statistics

Table 1 presents the demographic characteristics and prevalences of walking and cycling behaviors of the total, urban, semi-urban and rural sample. 35.9% of the total sample reported to walk for transportation daily and 23.7% stated to cycle for transportation daily. 53.8% of the participants reported to walk or cycle for recreation at least once a week.

**Table 1:** descriptive statistics

*Demographics*	Total sample N = 48879	Urban N = 15444	Semi-urban N = 17996	Rural N = 15439
age (years)	74.4 ± 6.7	74.5 ± 6.8	74.3 ± 6.6	74.3 ± 6.6

% female	55.7	56.7	55.3	55.2

% higher education	9.6	12.9	8.8	7.4

number of limitations	2.5 ± 2.6	2.5 ± 2.5	2.5 ± 2.6	2.6 ± 2.6

***Municipalities' characteristics***				

residential density (inh./km^2^)	577.0 ± 508.5	1112.6 ± 604.9	420.4 ± 86.9	223.7 ± 59.7

mean annual income (euro)	16438.6 ± 1728.8	17138.2 ± 1795.1	16468.6 ± 1718.5	15703.9 ± 1328.7

***Walking/cycling behavior***				

% daily walking for transportation	35.9	40.9	34.4	32.4

% daily cycling for transportation	23.7	20.6	25.7	24.5

% weekly walking or cycling for recreation	53.8	54.3	54	52.9

Means and frequencies of the environmental factors in the total sample and subgroups are presented in Table 2.

**Table 2:** Means and frequencies of the environmental factors in the total sample and subgroups

Environmental factors	Total sample	Urban	Semi-urban	Rural	< 75 years	≥ 75 years	Males	Females
***Access to services***								

Short distances to services (/5)^1^	3.9 ± 1.4	4.0 ± 1.4	3.9 ± 1.4	3.8 ± 1.5	4.0 ± 1.4	3.8 ± 1.5	4.0 ± 1.4	3.8 ± 1.5

Number of shops (/13)	9.7 ± 3.7	9.9 ± 3.5	9.5 ± 3.8	9.6 ± 3.8	9.8 ± 3.7	9.5 ± 3.7	9.7 ± 3.7	9.6 ± 3.7

Public transport (/5)	3.6 ± 1.1	3.8 ± 1.1	3.5 ± 1.1	3.4 ± 1.2	3.6 ± 1.1	3.6 ± 1.1	3.6 ± 1.1	3.5 ± 1.1

***Walking facilities***								

Presence of public toilets (%)^2^	57.5	55.0	57.9	59.7	56.7	58.6	56.0	58.7

Presence of benches (%)	61.0	62.5	60.2	60.6	61.4	60.6	61.2	60.7

Presence of crossings (%)	74.7	77.4	73.6	73.1	75.3	74.0	75.4	74.1

Condition of sidewalks (/5)	2.9 ± 1.3	2.7 ± 1.3	2.9 ± 1.2	3.0 ± 1.2	2.9 ± 1.2	2.9 ± 1.3	2.9 ± 1.2	2.8 ± 1.3

Absence of high ramps (/5)	4.6 ± 1.0	4.6 ± 1.0	4.6 ± 1.0	4.5 ± 1.0	4.6 ± 1.0	4.5 ± 1.0	4.6 ± 1.0	4.5 ± 1.0

***Safety***								

Traffic safety (%)	67.9	67.3	67.9	68.7	69.7	65.7	70.7	65.6

Feelings of unsafety (/8)	3.5 ± 1.1	3.6 ± 1.1	3.4 ± 1.1	3.4 ± 1.1	3.4 ± 1.0	3.5 ± 1.1	3.3 ± 1.0	3.6 ± 1.1

Street lighting (%)	81.4	83.9	80.6	79.8	81.8	80.9	81.7	81.2

***Aesthetics***								

Absence of decay (%)	92.9	90.7	93.5	94.6	92.4	93.6	92.8	93.1

Absence of noise (%)	80.9	78.7	80.9	83.2	80.1	81.9	80.1	81.6

Greenery (/5)	3.7 ± 1.0	3.7 ± 1.1	3.6 ± 1.0	3.7 ± 1.0	3.7 ± 1.0	3.6 ± 1.0	3.7 ± 1.0	3.6 ± 1.0

***Objective data***								

Public transportation subscriptions (% of older adults)	0.7 ± 0.6	1.0 ± 0.8	0.6 ± 0.4	0.6 ± 0.3	/	/	/	/

^1 ^For all factors higher scores represent more positive perceptions, except for feelings of unsafety

^2 ^Frequency percentages represent the percentage of participants perceiving the hypothesized positive aspect of the environmental factor to be present

### Analyses in the total sample

#### Relationships between area of residence and walking and cycling behaviors

Relationships for demographics, functional status and area of residence with walking and cycling are presented in Table 3.

**Table 3:** Predictors of walking and cycling in the total sample (OR, 95% CI) (multivariate)

	Daily walking for transportation	Daily cycling for transportation	Weekly walking or cycling for recreation
**Age**	1.04 (0.99, 1.09)	0.60 (0.57, 0.64)*	0.63 (0.60, 0.67)*

**Gender^1^**	0.79 (0.76, 0.83)*	0.69 (0.66, 0.73)*	0.75 (0.71, 0.78)*

**Education^2^**	1.11 (1.03, 1.19)*	0.68 (0.63, 0.75)*	1.05 (0.98, 1.14)

**Number of functional limitations**	0.85 (0.85, 0.86)*	0.81 (0.80, 0.82)*	0.77 (0.76, 0.78)*

**Area of residence:**			

**semi-urban^3^**	1.09 (0.96, 1.25)	1.08 (0.81, 1.44)	1.05 (0.90, 1.22)

**urban^3^**	1.43 (1.22, 1.67)*	0.81 (0.54, 1.21)	1.11 (0.94, 1.31)

**urban^4^**	1.32 (1.13, 1.54)*	0.72 (0.56, 0.92)*	1.04 (0.88, 1.22)

* *p *< 0.05

^1 ^reference group = males

^2 ^reference group = no higher education

^3 ^reference group = rural areas

^4 ^reference group = semi-urban areas

##### Daily walking for transportation

Urban respondents were 43% and 32% more likely to walk for transportation daily compared to rural and semi-urban respondents, respectively. No differences in likelihood of daily walking for transportation was observed for semi-urban versus rural residence.

##### Daily cycling for transportation

Urban residents were less likely to cycle for transportation daily compared to semi-urban residents. No differences in likelihood of daily cycling for transportation were observed for urban versus rural and semi-urban versus rural residents.

##### Weekly walking/cycling for recreation

No relationships between area of residence and weekly walking or cycling for recreation were observed.

#### Main effects of environmental factors in total sample

Main effects of the environmental factors for which no significant interaction effects were observed are presented in Table 4.

**Table 4:** Main effects of environmental factors in total sample

PA domain	Environmental factors	Main effect (OR, 95%C.I.)^1^
***Walking for transportation***	Short distances to services	1.19 (1.17, 1.21)*
	
	Public transport	1.13 (1.11, 1.16)*
	
	Quality of sidewalks	0.94 (0.92, 0.96)*
	
	Absence of high ramps	1.01 (0.99, 1.03)
	
	Feelings of unsafety	0.93 (0.91, 0.95)*
	
	Greenery	1.01 (0.99, 1.04)

***Cycling for transportation***	Number of shops	1.03 (1.02, 1.04)*
	
	Public transport	1.08 (1.05, 1.11)*
	
	Greenery	1.01 (0.98, 1.04)

***Walking/cycling for recreation***	Number of shops	1.02 (1.01, 1.03)*
	
	Feelings of unsafety	0.95 (0.92, 0.97)*

^1 ^Controlled for educational level and number of functional limitations

* *p *< 0.05

##### Daily walking for transportation

Perceived presence of services within short distances and satisfaction with public transport was positively related to walking for transportation. In contrary, a negative relationship was observed for quality of sidewalks and feelings of unsafety. No significant relationships were observed for absence of high ramps and satisfaction with greenery.

##### Daily cycling for transportation

Number of shops and satisfaction with public transport was positively related to daily cycling for transportation. No relationship was found for satisfaction with greenery.

##### Weekly walking/cycling for recreation

Number of shops was positively related to walking/cycling for recreation while feelings of unsafety were negatively related.

### Analyses in subgroups

#### Main effects for environmental factors with significant 3rd order interaction effects

Main effects in subgroups for environmental factors with significant 3rd order interaction effects are presented in Table 5.

**Table 5:** Third order interaction effects and main effects in subgroups

	Environmental factors	Interaction effect (β, SE)	Main effect in subgroups (OR, 95%C.I.)^1^											
		
		Environmental factor * urbanization * age * gender	Rural				Semi-urban				Urban			
			
			< 75		≥ 75		< 75		≥ 75		< 75		≥ 75	
			
			Males	Females	Males	Females	Males	Females	Males	Females	Males	Females	Males	Females
***Cycling for transportation***	Absence of decay	0.33 (0.14)	0.91 (0.66, 1.24)	1.43 (0.94, 2.18)	0.78 (0.47, 1.30)	0.64 (0.37, 1.11)	1.26 (0.91, 1.74)	1.16 (0.84, 1.59)	0.75 (0.49, 1.15)	0.96 (0.66, 1.41)	0.73 (0.57, 0.94)*	1.17 (0.85, 1.61)	1.13 (0.73, 1.75)	1.00 (0.53, 1.89)
	
	Absence of noise	0.37 (0.15)	1.13 (0.93, 1.38)	1.27 (1.01, 1.59)*	1.03 (0.76, 1.40)	0.85 (0.58, 1.25)	1.04 (0.87, 1.24)	1.14 (0.92, 1.41)	0.81 (0.61, 1.07)	0.87 (0.64, 1.20)	0.78 (0.64, 0.94)*	1.13 (0.92, 1.40)	1.17 (0.86, 1.61)	0.73 (0.50, 1.07)
	
	Public transportation subscriptions	-1.10 (0.34)	1.43 (0.74, 2.78)	1.73 (0.81, 3.70)	1.40 (0.70, 2.78)	2.07 (0.99, 4.34)	1.49 (0.92, 2.40)	1.26 (0.76, 2.08)	1.29 (0.78, 2.13)	1.41 (0.76, 2.60)	0.83 (0.52, 1.35)	1.04 (0.54, 2.02)	0.91 (0.55, 1.48)	0.73 (0.46, 1.15)

***Walking/cycling for recreation***	Presence of public toilets	0.38 (0.17)	0.95 (0.81, 1.11)	0.99 (0.84, 1.16)	1.14 (0.93, 1.39)	0.84 (0.70, 1.01)	1.05 (0.90, 1.21)	0.99 (0.86, 1.15)	0.88 (0.73, 1.06)	0.84 (0.71, 1.00)*	0.93 (0.79, 1.10)	0.90 (0.77, 1.05)	0.88 (0.72, 1.08)	0.89 (0.75, 1.06)
	
	Greenery	-0.30 (0.15)	1.04 (0.95, 1.13)	0.97 (0.88, 1.07)	0.93 (0.82, 1.05)	1.04 (0.93, 1.17)	1.06 (0.97, 1.15)	1.05 (0.97, 1.15)	1.04 (0.93, 1.17)	1.07 (0.97, 1.18)	1.01 (0.92, 1.10)	1.05 (0.96, 1.15)	0.96 (0.86, 1.07)	0.90 (0.81, 0.99)*

^1 ^Controlled for educational level and number of functional limitations

* *p *< 0.05

##### Cycling for transportation

For absence of decay a negative relationship with cycling for transportation was only observed in the urban < 75 years old males. Absence of noise was positively related to cycling for transportation in rural < 75 years old females, but negatively related in urban < 75 years old males. No significant relationships were found for public transportation subscriptions. However, the ORs were lower in urban compared to rural and semi-urban subgroups and highest in rural female subgroups.

##### Weekly walking/cycling for recreation

A negative relationship between presence of public toilets and walking/cycling for recreation was observed in semi-urban ≥ 75 years old females. A negative relationship between satisfaction with greenery and walking for recreation was observed in urban ≥ 75 years old females. For both presence of public toilets and satisfaction with greenery, no significant relationships with walking/cycling for recreation were observed in other urbanization*age*gender subgroups.

#### Main effects for environmental factors with significant 2nd order interaction effects

Main effects in subgroups for environmental factors with significant 2nd order interaction effects are presented in Table 6.

**Table 6:** Second order interaction effects and main effects in subgroups

PA domain	Environmental factors	Interaction effect (β, SE)	Main effect in subgroups (OR, 95%C.I.)^1^					
		Environmental factor * urbanization * age	**Rural**		**Semi-urban**		**Urban**	
			
			**< 75**	**≥ 75**	**< 75**	**≥ 75**	**< 75**	**≥ 75**
	
***Walking for transportation***	Number of shops	0.04 (0.02)	1.02 (1.01, 1.04)*	1.01 (0.99, 1.02)	1.05 (1.04, 1.07)*	1.04 (1.02, 1.05)*	1.03 (1.01, 1.04)*	1.05 (1.04, 1.07)*
	
	Public transportation subscriptions	0.49 (0.17)	1.00 (0.75, 1.32)	0.79 (0.58, 1.07)	0.79 (0.56, 1.13)	0.85 (0.63, 1.15)	1.36 (1.13, 1.62)*	1.39 (1.12, 1.73)*

***Walking/cycling for recreation***	Short distances to services	-0.14 (0.06)	1.07 (1.03, 1.11)*	1.12 (1.07, 1.17)*	1.12 (1.08, 1.16)*	1.06 (1.02, 1.11)*	1.04 (1.00, 1.09)*	1.02 (0.97, 1.06)
	
	% public transportation subscriptions	0.67 (0.21)	1.37 (0.90, 2.07)	0.91 (0.59, 1.40)	1.34 (0.88, 2.05)	1.18 (0.86, 1.63)	0.92 (0.77, 1.11)	1.13 (0.91, 1.39)

		Environmental factor * urbanization * gender	**Rural**		**Semi-urban**		**Urban**	
			
			**Males**	**Females**	**Males**	**Females**	**Males**	**Females**
***Cycling for transportation***	Presence of public toilets	-0.27 (0.11)	1.07 (0.93, 1.23)	1.00 (0.87, 1.16)	1.09 (0.97, 1.23)	0.98 (0.87, 1.11)	0.98 (0.86, 1.12)	0.86 (0.74, 1.00)
	
	Presence of benches	-0.25 (0.11)	1.02 (0.89, 1.17)	1.21 (1.04, 1.41)*	1.09 (0.97, 1.24)	0.93 (0.82, 1.06)	1.06 (0.92, 1.23)	1.01 (0.86, 1.19)
	
	Presence of street lighting	-0.28 (0.10)	0.85 (0.73, 1.00)	1.31 (1.07, 1.60)*	0.89 (0.76, 1.04)	0.97 (0.83, 1.14)	1.16 (0.95, 1.41)	1.00 (0.82, 1.23)

***Walking/cycling for recreation***	Public transport	0.15 (0.08)	1.09 (1.02, 1.16)*	1.05 (1.00, 1.12)	1.11(1.05, 1.18)*	1.12 (1.06, 1.18)*	1.07 (1.00, 1.14)*	1.09 (1.02, 1.15)*
	
	Presence of crossings	-0.17 (0.08)	0.94 (0.82, 1.07)	1.00 (0.88, 1.13)	1.03 (0.90, 1.18)	0.95 (0.84, 1.07)	1.05 (0.90, 1.21)	0.98 (0.85, 1.13)

		Environmental factor * age * gender	**< 75**			**≥ 75**		
			
			**Males**	**Females**		**Males**	**Females**	
	
***Walking for transportation***	Absence of decay	-0.21 (0.10)	0.71 (0.61, 0.83)*	0.70 (0.61, 0.81)*		0.74 (0.59, 0.92)*	0.82 (0.67, 1.00)	
	
	Absence of noise	-0.23 (0.11)	0.87 (0.79, 0.97)*	0.73 (0.66, 0.81)*		0.81 (0.71, 0.93)*	0.77 (0.68, 0.87)*	

***Cycling for transportation***	Presence of public toilets	-0.79 (0.15)	1.06 (0.97, 1.16)	0.93 (0.84, 1.03)		1.04 (0.91, 1.18)	1.00 (0.86, 1.16)	
	
	Presence of benches	-0.73 (0.13)	1.10 (1.00, 1.20)	1.05 (0.95, 1.16)		0.96 (0.84, 1.10)	1.01 (0.87, 1.18)	
	
	Presence of crossings	-0.81 (0.14)	1.03 (0.93, 1.14)	1.00 (0.89, 1.12)		1.08 (0.93, 1.25)	0.96 (0.81, 1.13)	
	
	Traffic safety	-0.76 (0.11)	0.77(0.70, 0.85)*	0.84 (0.76, 0.94)*		0.79 (0.69, 0.90)*	0.74 (0.64, 0.86)*	
	
	Presence of street lighting	-0.79 (0.12)	1.00 (0.88, 1.12)	1.16 (1.03, 1.30)*		0.87 (0.74, 1.03)	0.92 (0.76, 1.11)	

***Walking/cycling for recreation***	Presence of benches	-0.38 (0.10)	1.01 (0.92, 1.12)	1.02 (0.93, 1.12)		1.00 (0.89, 1.13)	0.89 (0.81, 0.99)*	
	
	Presence of crossings	-0.44 (0.09)	1.05 (0.94, 1.16)	1.03 (0.93, 1.14)		0.97 (0.84, 1.12)	0.90 (0.81, 1.00)	
	
	Traffic safety	-0.54 (0.11)	0.84 (0.76, 0.92)*	0.86 (0.79, 0.95)*		0.86 (0.77, 0.97)*	0.67 (0.61, 0.74)*	
	
	Presence of street lighting	-0.34 (0.10)	1.00 (0.90, 1.11)	1.19 (1.05, 1.35)*		0.93 (0.80, 1.07)	1.00 (0.88, 1.14)	
	
	Absence of decay	-0.41 (0.10)	1.07 (0.91, 1.26)	0.85 (0.71, 1.01)		0.72 (0.58, 0.90)*	0.79 (0.65, 0.95)*	
	
	Absence of noise	-0.39 (0.10)	1.02 (0.93, 1.13)	0.95 (0.85, 1.06)		0.85 (0.75, 0.97)*	0.86 (0.76, 0.98)*	

^1 ^Controlled for educational level and number of functional limitations

* *p *< 0.05

##### Walking for transportation

In all urbanization*age subgroups, a positive relationship with walking for transportation was found for number of shops, except in rural ≥ 75 year olds. For percentage public transportation subscriptions a positive relationship was found solely in the urban subgroups with the largest OR in the ≥ 75 year olds.

For absence of decay a negative relationship with walking for transportation was observed in all age*gender subgroups, except in the ≥ 75 years old females. For absence of noise, a negative relationship with walking for transportation was observed in all subgroups. In females this relationship was strongest in the < 75 year olds whereas in males this relationship was strongest in the ≥ 75 year olds.

##### Cycling for transportation

Although a significant urbanization*gender interaction effect was found for presence of public toilets, no significant relationships with cycling for transportation were observed in any of the subgroups. However, the ORs were highest for rural and semi-urban males and lowest for urban females. Presence of benches and street lighting was positively related to cycling for transportation in rural females, whereas in other urbanization*gender subgroups no significant relationship was found.

In none of the age*gender subgroups, presence of public toilets was significantly related to cycling for transportation, although a significant interaction effect was found. The observed ORs were higher in males compared to females, with the highest value in < 75 years old males. Similarly, no significant relationships were observed between presence of benches and cycling for transportation in any of the age*gender subgroups. However, ORs were higher in < 75 year olds compared to ≥ 75 year olds and highest in < 75 years old males. For perceived presence of crossings no significant relationships with cycling for transportation were observed, despite the significant interaction effect. The ORs were higher in males compared to females and highest in ≥ 75 years old males. A negative relationship was observed for traffic safety in the four subgroups. In the youngest age group this relationship was stronger in males while in the oldest age group this relationship was stronger in females. Presence of street lighting was significantly positively related to cycling for transportation in the < 75 years old females but unrelated in the other age*gender subgroups.

##### Weekly walking/cycling for recreation

In all urbanization*age subgroups perceived presence of services within short distances was positively related to walking/cycling for recreation, except in urban ≥ 75 year olds. For % public transportation subscriptions, relationships were non-significant in all urbanization*age subgroups despite a significant interaction effect. However, ORs were higher for rural and semi-urban < 75 year olds compared to other urbanization*age subgroups.

Satisfaction with public transport was positively related to walking/cycling for recreation in all urbanization*gender subgroups, except in the rural female subgroup. For presence of crossings, no significant relationships with walking/cycling for recreation were observed but the ORs were slightly higher for the semi-urban and urban males compared to other subgroups.

Presence of benches was negatively related to walking/cycling for recreation in the ≥ 75 years old females. No significant relationships with walking/cycling for recreation were found in the other age*gender subgroups. For presence of crossings no significant relationships with walking/cycling for recreation were observed, although the interaction effect was significant. However, the OR was markedly lower in ≥ 75 years old females compared to other subgroups. Traffic safety was negatively related in the four age*gender subgroups with the lowest OR for the ≥ 75 years old females. The presence of street lighting was positively related in the < 75 years old females while no significant relationship was found in other age*gender subgroups. For absence of decay, a negative relationship with walking for recreation was found in both ≥ 75 years old subgroups. This relationship was strongest in males. Absence of noise was negatively related to walking/cycling for recreation in both ≥ 75 years old subgroups. No relationship was observed in < 75 years old males and females.

#### Main effects for environmental factors with significant 1st order interaction effects

Main effects in subgroups for environmental factors with significant 1st order interaction effects are presented in Table 7.

**Table 7:** First order interaction effects and main effects in subgroups

PA domain	Environmental factors	Interaction effect (β, SE)	**Main effect in subgroups **(OR, 95%C.I.)^1^		
		Environmental factor * urbanization	**Rural**	**Semi-urban**	**Urban**
	
***Walking for transportation***	Presence of crossings	0.28 (0.08)	0.86 (0.78, 0.94)*	1.11 (1.03, 1.21)*	1.03 (0.93, 1.13)

		Environmental factor * age	**< 75 years**	**≥ 75 years**	
	
***Walking for transportation***	Presence of public toilets	0.17 (0.08)	0.80 (0.75, 0.85)*	0.83 (0.77, 0.89)*	
	
	Presence of benches	0.18 (0.08)	0.98 (0.92, 1.04)	0.99 (0.92, 1.07)	
	
	Presence of crossings	0.17 (0.08)	0.98 (0.92, 1.05)	1.03 (0.94, 1.12)	
	
	Presence of street lighting	0.20 (0.09)	1.08 (1.00, 1.16)*	1.10 (1.01, 1.21)*	

		Environmental factor * gender	**Males**	**Females**	
	
***Walking for transportation***	Traffic safety	0.19 (0.09)	0.83 (0.77, 0.89)*	0.89 (0.83, 0.95)*	
	
	Presence of street lighting	0.17 (0.07)	1.03 (0.95, 1.12)	1.14 (1.05, 1.24)*	

***Cycling for transportation***	Short distances to services	0.10 (0.04)	1.08 (1.05, 1.11)*	1.14 (1.11, 1.18)*	
	
	Feelings of unsafety	-0.11 (0.05)	0.97 (0.93, 1.01)	0.94 (0.90, 0.98)*	

^1 ^Controlled for educational level and number of functional limitations

* *p *< 0.05

##### Daily walking for transportation

The presence of crossings was unrelated to walking for transportation in urban areas. However, in semi-urban areas it was positively related and in rural areas it was negatively related to walking for transportation.

Regarding interactions with age, presence of public toilets was negatively related to walking for transportation in both subgroups, but the relationship was slightly stronger in the < 75 year olds. Although a significant interaction effect with age was found for presence of benches and crossings, no significant relationships with walking for transportation were observed. The ORs were somewhat lower in the < 75 years olds. The presence of street lighting was positively related to walking for transportation in both age groups with a somewhat stronger relationship in the ≥ 75 years olds.

For traffic safety a negative relationship with walking for transportation was observed in both males and females. This relationship appeared to be stronger in males compared to females. Presence of street lighting was unrelated to walking for transportation in males but positively related in females.

##### Daily cycling for transportation

Perceived presence of services within short distances was positively related to cycling for transportation. This relationship was stronger in females compared to males. For feelings of unsafety, no significant relationship with cycling for transportation was observed in males while a negative relationship was observed in females.

## Discussion

This is the first study investigating relationships between urban, semi-urban and rural residence and older adults' walking and cycling. Furthermore, the relationship between the physical environment and walking and cycling behaviors and its possible moderators were explored.

35.9% of the participants reported to *walk for transportation *daily, however urban participants were 43% and 32% more likely to walk for transportation daily compared to rural and semi-urban older adults, respectively. This is in concordance with findings in Australian [27] and Flemish adults [28] and might be explained by shorter distances to shops and services in urban areas. The latter is supported by the positive relationship between short distances to services and daily walking for transportation. However, no marked difference between urban, semi-urban and rural participants is observed in their ratings on the 'short distances to services' - measure (Table 2). Possibly, urban, semi-urban and rural residents perceive distances differently. A higher number of neighborhood shops was also associated with more transportation walking in all subgroups except for rural ≥ 75 year olds. Hence, our findings support results from two previous studies on the application of the 'walkability' concept in older adults. Frank and colleagues [14] found older adults living in objectively defined high-walkable neighborhoods to be twice as likely to have walked for transportation in the past 2 days than those in low-walkable neighborhoods. In another US study older adults living in objectively defined high-walkable neighborhoods accumulated 30 minutes/week more transport activity (walking and cycling) compared with those living in low-walkable neighborhoods [15].

An important destination to walk to and from appears to be public transit. Greater satisfaction with public transportation was associated with more walking for transportation. In addition, a higher percentage of public transportation subscriptions was related to more transportation walking in urban subgroups. Having access to public transit might enable older adults to reach destinations situated further away by a shortened walk to a bus or rail stop. Investigating public transit characteristics in British older adults, Su and colleagues [12] reported less time between bus services and higher bus stops density to be associated with more walking for shopping purposes.

A higher score on the 'Elderly Feelings of Unsafety scale' was associated with lower prevalences of daily walking for transportation. This is in accordance with a previous study which reported perceived neighborhood problems (e.g. vandalism) to be related to less transportation walking in US older adults [29]. However in another study in US older adults, Shigematsu and colleagues reported no relationship for perceived safety from crime. These discrepancies in findings might be explained by the questionnaires used to asses perceptions of safety [30]. Presence of street lighting, a factor possibly influencing feelings of unsafety, was associated with more daily walking for transportation in females but not in males. This relationship was also somewhat stronger in the oldest age group.

No relationship for presence of crossings was found in urban residents, whereas a positive relationship was found in semi-urban residents and a negative relationship in rural residents. A possible explanation for the importance of crossings in semi-urban areas is the location of busy arterial roads, which necessitates the presence of crossings in order to cross them safely, in these areas. The negative relationship between presence of crossings and walking for transportation observed in rural areas was unexpected and possible reasons are unclear.

In a Dutch study [13] the objectively measured absence of high ramps, presence of benches and trees was unrelated to a street's use for transportation walking. Similarly, the perceived absence of high ramps, presence of benches and satisfaction with greenery was unrelated to daily walking for transportation in the present study.

Unexpected negative relationships for traffic safety, presence of public toilets, quality of sidewalks and absence of noise and decay were observed. A possible explanation is the cross-sectional nature of this study as participants who walk daily encounter and suffer from these problems more frequently and might report them more easily. Furthermore, destinations (e.g. shops, post offices...) are often located in busy shopping streets which might explain that more walking for transportation was associated with lower perceptions of absence of decay and noise.

Almost one quarter of the participants reported to *cycle for transportation *daily. Urban residents were less likely to cycle for transportation daily compared to semi-urban residents. No differences in likelihood of daily cycling were found for rural versus semi-urban and rural versus urban residents. These findings suggest that when distances are longer older adults rather cycle instead of walk, which seems obvious as greater distances are easier and faster covered cycling compared to walking. However, in rural areas distances might be too large in order to bridge them by cycling. Perceived short distances to services was indeed related to daily cycling for transportation, but the relationship was stronger in females compared to males. Possibly, lower physical fitness levels in females compared to males [31] and consequently more difficulties in overcoming greater distances explain these gender discrepancies. Number of shops in the neighborhood was also related to a higher likelihood of daily cycling for transportation.

Higher satisfaction with public transportation was related to more cycling for transportation. Again, public transportation might enable older adults to bridge larger distances compared to cycling alone. Investigating the relationship between types of public transportation and the transport mode to reach them, cycling was found to be more common to faster and high quality types of public transportation (e.g. trains) compared to slower and lower quality types of public transportation (e.g. local buses) [32]. Walking was reported to be most common when the railway station is situated within 1.5 km from the participants' home. Cycling to the station was most popular when located between 1.5 and 3.5 km away [33]. In the present study, the relationship between percentage of public transportation subscriptions did not reach significance in any of the subgroups, although the ORs were large, especially in rural areas. If these trends are confirmed in future studies, providing a well-accessible public transport network seems a good strategy to promote cycling for transportation. More studies on relationships between characteristics of public transit and older adults' active transportation are needed.

Feelings of unsafety were related to a decreased likelihood of daily cycling for transportation in females but not in males. Related to this, presence of street lighting increased the likelihood of daily cycling in < 75 years old females and rural females but not in other subgroups. Apparently, with regard to cycling for transportation women are more susceptible to feelings of unsafey. Providing adequate street lighting might be one solution to overcome this problem.

Traffic safety was negatively related to daily cycling for transportation which might again be explained by the cross-sectional nature of this study. Older adults who cycle daily might experience traffic concerns frequently and therefore report them more easily.

The presence of public toilets, benches, crossings, satisfaction with greenery was found to be unrelated to cycling for transportation. Absence of decay and noise were not related to cycling for transportation in most subgroups. However for both, a negative relationship was found in urban < 75 years old males and a positive relationship was observed in rural < 75 years old females for absence of noise. The reasons for these inconsistent findings between subgroups are unclear.

About half of the participants reported *to walk or cycle for recreation *weekly. Walking and cycling for recreation was unrelated to area of residence. This is in line with findings for recreational walking in Australian adults [27]. One might expect recreational walking and cycling to be more common in the green and quiet rural areas. However, results of the present study suggest that urban older adults walk or cycle for recreation in city centers and shopping streets. This is supported by the positive relationships for number of shops and short distances to services. However, Van Dyck and colleagues [28] reported more recreational walking but less cycling in urban compared to rural neighborhoods in Flemish adults. These opposite relationships might have resulted in no relationship for the combination of recreational walking and cycling in the present study.

Feelings of unsafety were associated with lower prevalences of weekly walking/cycling for recreation. This finding supports results from a longitudinal study in the US which showed perceived general safety to be related to less decline in older adults' recreational walking at 12 months follow-up [34]. However, perceived general walking safety was unrelated to older adults' recreational walking in two other US studies [35,36]. These three studies used a general safety measure whereas in the present study it specifically focused on safety from crime. Furthermore, it was shown in the present study that this relationship between safety from crime and recreational walking/cycling holds true for both males and females, < 75 year olds and ≥ 75 years old and in urban, semi-urban as well as rural areas.

In summary, no clear patterns emerged for environmental factors other than access to destinations and feeling of unsafety. Possibly, the presence of one 'favorable' environmental factor might not be sufficient to influence walking or cycling behaviors. This is supported by the strong relationship between walking and cycling for transportation and area of residence, which is characterized by a specific combination of environmental factors. Sallis and colleagues [37] reported that at least 4 favorable environmental factors needed to be present to find a significant relationship with adults' PA.

A first strength of this study is the large sample size, which enabled us to investigate interaction effects and create subgroups. Secondly, the sample included urban, semi-urban and rural residents while previous studies primarily focused on urban residents. Lastly, the focus on specific PA domains and especially on cycling behaviors, which had not been studied before in this population, certainly adds to the value of this study. However, aggregating walking and cycling for recreation might have obscured relationships with these behaviors. A second limitation is the absence of information on the psychometrics of PA measures used in the present study. Furthermore, only frequency of walking and cycling was assessed and walking and cycling for transportation were expressed as daily habits while walking/cycling for recreation was assessed as a weekly habit. Consequently, caution is needed when comparing results for transportation and recreational walking and cycling. Furthermore, given the relatively high prevalences of walking and cycling in our sample, our findings might not be completely applicable to other contexts. Thirdly, some potentially important environmental factors were not assessed (e.g. presence of cycling facilities). A last limitation is the cross-sectional design which prohibits us from drawing conclusions about causality.

## Conclusions

Area of residence was found to be related to older adults' walking and cycling for transportation but not to walking and cycling for recreation. Urban participants were more likely to walk for transportation daily compared to their semi-urban and rural counterparts. Daily cycling for transportation was less prevalent among urban participants compared to semi-urban participants.

Access to destinations appeared to be a correlate of both walking and cycling for transportation and recreation across all demographic subgroups. A well-connected public transportation system might offer a solution to reach utilitarian/recreational destinations located beyond walking/cycling distance. In addition, feelings of unsafety were associated with lower rates of walking for transportation and walking/cycling for recreation in all demographic subgroups and cycling for transportation in females. Our results point to the importance of adequate street lighting, especially in females, which might influence feelings of unsafety. Further research is needed in order to inform policy makers and urban planners on how to design neighborhoods that optimally promote older adults' walking and cycling.

## Competing interests

The authors declare that they have no competing interests.

## Authors' contributions

DV, NDW, LDD, TB and SD developed the design of the Belgian Aging Studies and were responsible for data collection. JVC, PC, IDB, VVH, DV, LDD and BD designed the protocol for this (sub)study. JVC performed the statistical analyses and drafted the manuscript. BD helped during the statistical analyses. JVC, PC, IDB, VVH, DV, LDD and BD critically revised and helped to draft the manuscript. All authors read and approved the final manuscript.

### Funding

The first author was supported by a grant from the Research Council of the Vrije Universiteit Brussel.

## Pre-publication history

The pre-publication history for this paper can be accessed here:

http://www.biomedcentral.com/1471-2458/12/142/prepub
